# *Octodon degus* laboratory colony management principles and methods for behavioral analysis for Alzheimer’s disease neuroscience research

**DOI:** 10.3389/fnagi.2024.1517416

**Published:** 2025-01-20

**Authors:** B. Maximiliano Garduño, Todd C. Holmes, Robert M. J. Deacon, Xiangmin Xu, Patricia Cogram

**Affiliations:** ^1^Department of Anatomy and Neurobiology, School of Medicine, University of California, Irvine, Irvine, CA, United States; ^2^Department of Physiology and Biophysics, School of Medicine, University of California, Irvine, Irvine, CA, United States; ^3^The Center for Neural Circuit Mapping, University of California, Irvine, Irvine, CA, United States; ^4^Department of Ecological Sciences, Faculty of Sciences, Institute of Ecology and Biodiversity, Universidad de Chile, Santiago, Chile; ^5^Institute for Memory Impairments and Neurological Disorders, University of California, Irvine, Irvine, CA, United States

**Keywords:** *Octodon degus*, husbandry, standard operating procedures, Alzheimer’s disease, animal models

## Abstract

The Chilean degu (*Octodon degus*) is a medium sized, long-lived rodent with traits that make them a natural model for neuroscience research. Their social behaviors, diurnality, and extended developmental time course, when compared to other rodents, make them useful for social behavioral, chronobiology, and developmental research. Lab-kept degus have a long lifespan (5–8 years) and may naturally develop age-related diseases that resemble Alzheimer’s disease. While there is significant interest in using the *Octodon degus* for neuroscience research, including aging and Alzheimer’s disease studies, laboratory management and methods for degus research are currently not standardized. This lack of standardization potentially impacts study reproducibility and makes it difficult to compare results between different laboratories. Degus require species-specific housing and handling methods that reflect their ecology, life history, and group-living characteristics. Here we introduce major principles and ethological considerations of colony management and husbandry. We provide clear instructions on laboratory practices necessary for maintaining a healthy and robust colony of degus for Alzheimer’s disease neuroscience research towards conducting reproducible studies. We also report detailed procedures and methodical information for degu *Apoe* genotyping and ethologically relevant burrowing behavioral tasks in laboratory settings.

## 1 Introduction

**Figure 1:**
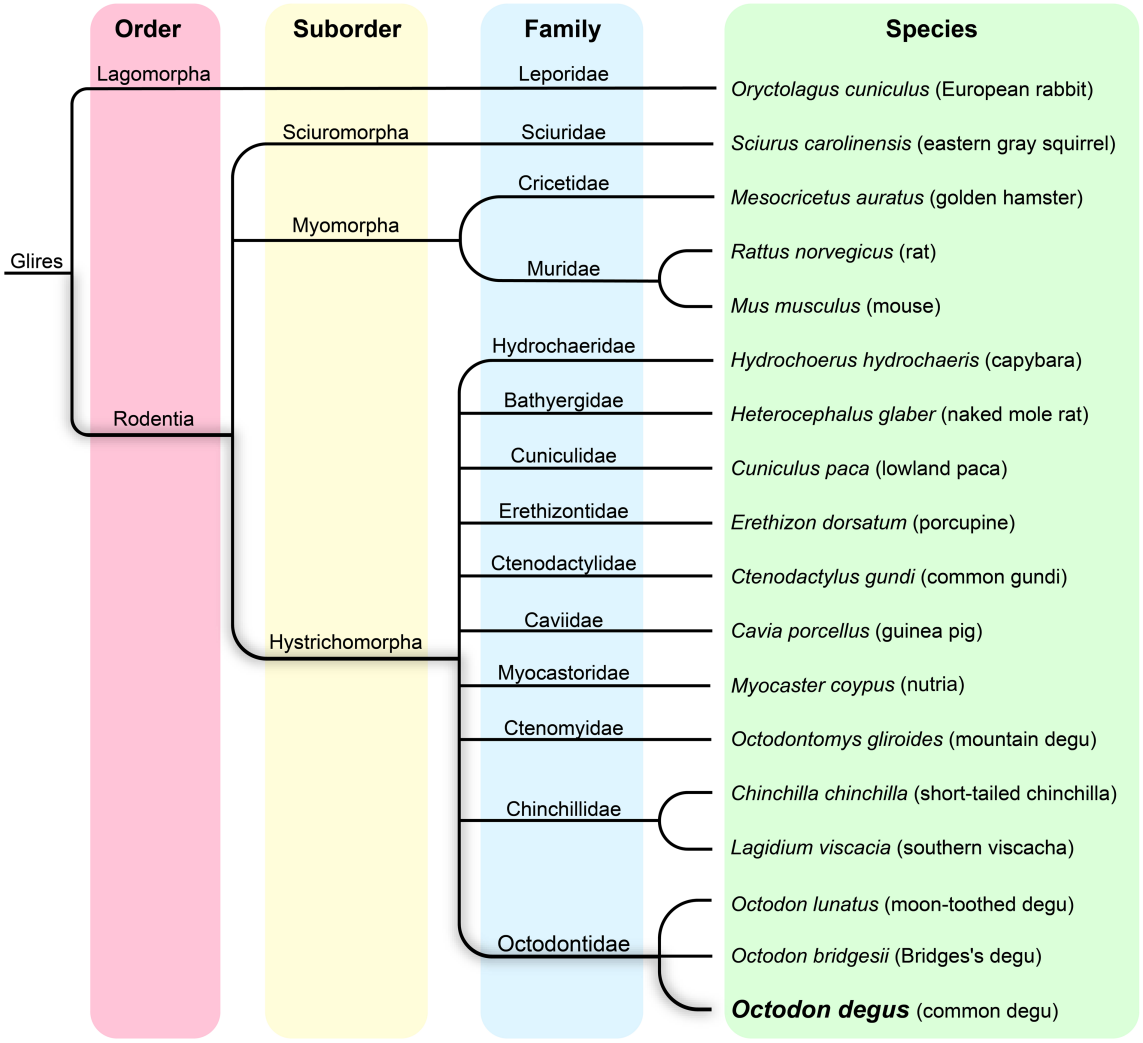
Phylogenetic tree of the *Octodon degus*, related hystricomorphs, and other representative rodent species.

The *Octodon degus*, commonly known as the degu, is a herbivorous caviomorph rodent endemic to Chile that dwells in semifossorial habitats distributed from 28° 30′ to 34° S (Meserve and Glanz, 1978; Contreras et al., 1987; Muñoz-Pedreros and Yáñez, 2000) in north-to-central Chile. Within the Octodontidae family, the degu belongs to the Octodon genus which contains several other species (Figure 1). They share similar physical characteristics and social behaviors, suggesting a close evolutionary relationship, although degus are the only diurnal species within the genus. The Octodontidae family is part of the Hystricomorpha suborder, a diverse group of rodents that also includes guinea pigs, chinchillas, and capybaras; rats and mice are part of the Myomorpha suborder of rodents (Figure 1).

**Figure 2:**
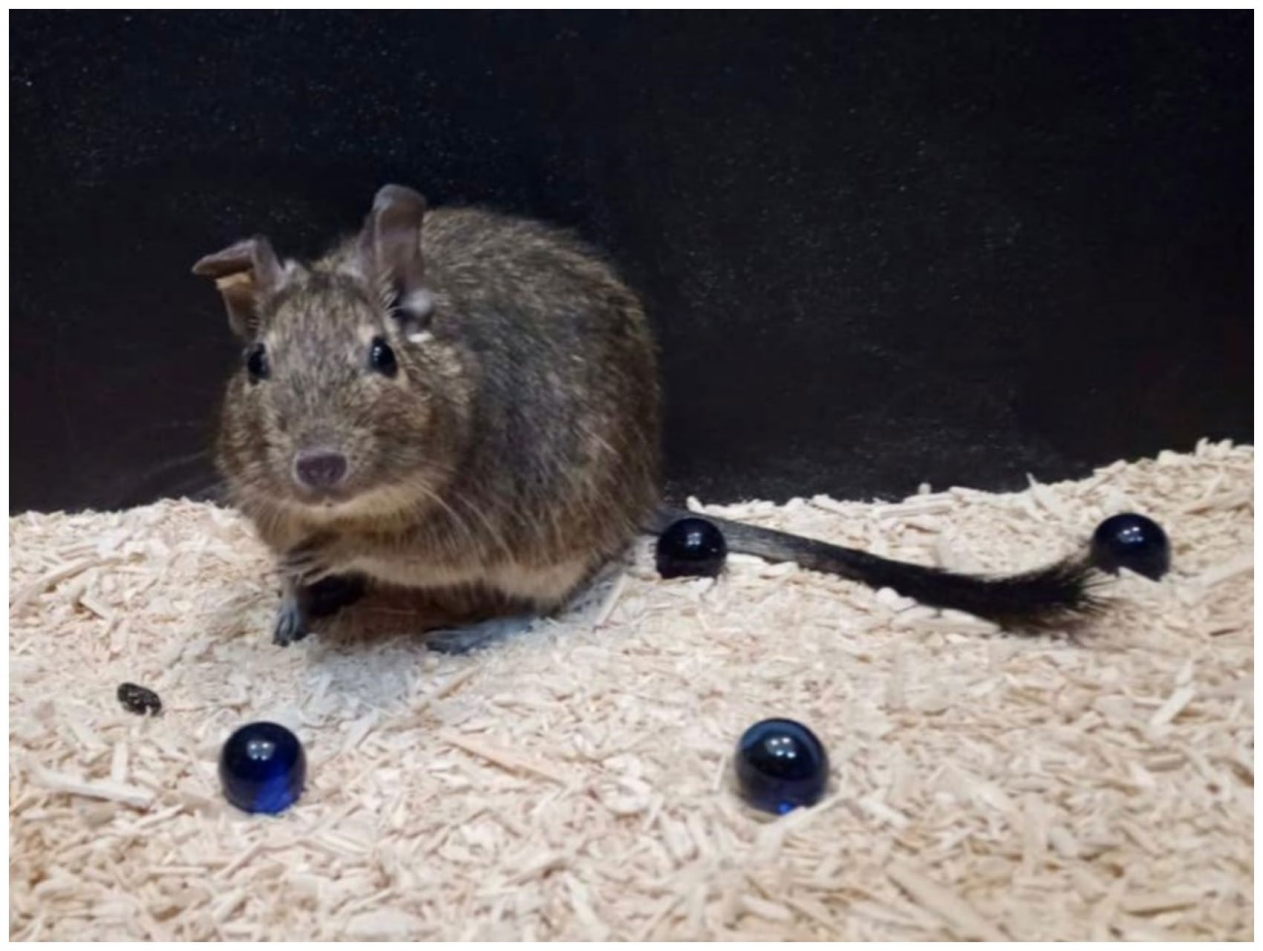
Photo of an adult degu conducting a marble burying test showing the characteristic trumpet tail.

Wild populations of degus occupy varied geographic habitats with strong gradients of climatic and terrain severity in Chile. Unlike mice and rats, degus are precocious rodents born with a full coat of fur and open eyelids. Their coat is dark grey to brown in color, lighter ventrally, and there is a characteristic tuft of hair at the end of their tail, for which they are sometimes referred to as “trumpet tailed rats” (Figure 2). Their weight varies between 170–300 g in adults with a body length of 125–195 mm (Woods and Boraker, 1975). Being a prey species, they seldom live longer than 2 years in the wild, while in captivity they live up to 5–8 years (Ardiles et al., 2013; Palacios and Lee, 2013). Their hearing and vision are excellent (Ardiles et al., 2013; Márquez et al., 2024). As diurnal rodents, their retina possesses two types of cones in addition to rods, sensitive to ultraviolet (UV) and green light (M). The degu’s paler ventral coat, as well as their fresh urine, are both UV reflective, which are thought to have communicative and scent marking roles (Ardiles et al., 2013). Degus are a highly vocal species, with up to 17 different calls identified in previous studies (Tokimoto and Okanoya, 2004).

Degus have proven to be valuable animal models in several areas of neuroscience (Long, 2007; Hagenauer and Lee, 2008; Lee et al., 2009; Vega-Zuniga et al., 2013; Akers et al., 2014; Bauer et al., 2019; Lidhar et al., 2021; Garduño et al., 2024) and human disease research (Spear et al., 1984; Opazo et al., 2004; Edwards, 2009; Homan et al., 2010; Jekl et al., 2011b; Chang et al., 2020; Švara et al., 2020; Chang et al., 2021). Among these emerged Alzheimer’s disease (AD), the most common form of progressive dementia that affect millions of people around the world (Alzheimer’s Association Report, 2024). The presence of AD-like neuropathology, similar to what is seen in human AD, was first reported in degus by Inestrosa et al. (2005). This and further studies identified the presence of intra- and extracellular amyloid-ß plaques, phosphorylated tau, neuroinflammation, neuronal death, and circuit hyperactivity in the degu brain (Cisternas et al., 2018; Tan et al., 2022). Unlike current AD mouse models, these pathological traits manifest naturally in degus without any form of genetic engineering intervention, making them an attractive model to investigate the prevalent form of the condition: sporadic AD (Masters et al., 2015; Cogram et al., 2024). Coupled with their highly social nature and susceptibility to metabolic and cardiovascular diseases, degus provide expanded value as a model to investigate AD comorbidities and psychosocial abnormalities that contribute to the onset and progression of this debilitating multifactorial condition (Homan et al., 2010; Colby et al., 2012; Cisternas et al., 2018; Polis and Samson, 2024).

Despite these compelling initial findings, AD degu research remains relatively preliminary. Different research groups have reported conflicting results, with several studies failing to find AD-like neuropathology in lab inbred degus (Steffen et al., 2016; Bourdenx et al., 2017). We hypothesize the root of these inconsistencies could be due to genetic background differences between laboratory inbred colonies and more genetically diverse outbred degus. Other likely contributing factors include relatively small sample sizes and the absence of behavioral screening.

To resolve these inconsistencies and to determine whether degus are indeed a good natural model of sporadic AD, our studies focus on outbred, genetically diverse degus that are stocked from wild-caught animals in Chile (Deacon et al., 2015; Hurley et al., 2022; Tan et al., 2022). As AD is behaviorally characterized by cognitive impairments, we first screened degus by assaying the presence or absence of cognitive deficits using an ethologically relevant burrowing behavior paradigm. Burrowing performance requires hippocampal function and is a more relevant species-specific behavior assay for assessing cognitive status in burrowing rodents like degus (Deacon, 2009; Deacon et al., 2015). Our data show that about 1/3 of outbred adult degus show burrowing deficits (Deacon et al., 2015). This is critical as AD is sporadic: it does not occur in all humans but does appear in a subpopulation of aged humans. We compared correlative neuropathology between these two groups of degus that were pre-screened based on behavioral performance: “AD-like” degus with impaired burrowing performance versus age-matched “Non-AD” control degus with intact burrowing performance. The cognitively impaired degus show AD-like neuropathological features, while age-matched degus that are not behaviorally impaired do not exhibit them. Our findings, taken together, show spontaneous AD-like correlative phenotypes in cognitive performance and neuropathology in aged, outbred degus (Tan et al., 2022). This supports the proposition that genetically diverse aged degus are a useful and practical model of natural sporadic AD and highlights the importance of ethologically relevant behavioral screening.

Below, we first review and summarize the relevant degu ecology, behavior, and physiology that are reported in earlier studies. We consider the unique characteristics of the degu, their natural habitat, and address how these considerations should guide conducting research on degus. Then, based on our own successful establishment of outbred degu colonies, we provide specific instructions and best practices for degu colony management in laboratory and experimental settings. In addition, we report detailed procedures and methodical information for performing *Apoe* genotyping and the AD-related burrowing behavior test in laboratory settings.

## 2 Materials and methods

### 2.1 Ecology, behavior, and physiology of degus in the wild

#### 2.1.1 Natural habitat

Most degus prefer open habitats and inhabit the matorral plains of central Chile, where they are plentiful (Ebensperger et al., 2012). They construct underground burrows to live and hide in during the night, and go above ground to forage during the day (Ebensperger et al., 2004). Their natural open or scrub environment partially explains their social nature, as there is “safety in numbers.” Larger groups allow their individuals to devote less time to vigilant guarding, freeing more time for foraging (Ebensperger et al., 2006a).

Degus are herbivorous. Grasses, leaves, occasional roots, and seeds form most of their diet. Sugary foods are scarce in their natural environment, which is thought to have placed evolutionary pressure on the degu’s carbohydrate metabolism and consequentially made them highly sensitive to dietary sugar (Nishi and Steiner, 1990; Opazo et al., 2004).

**Figure 3:**
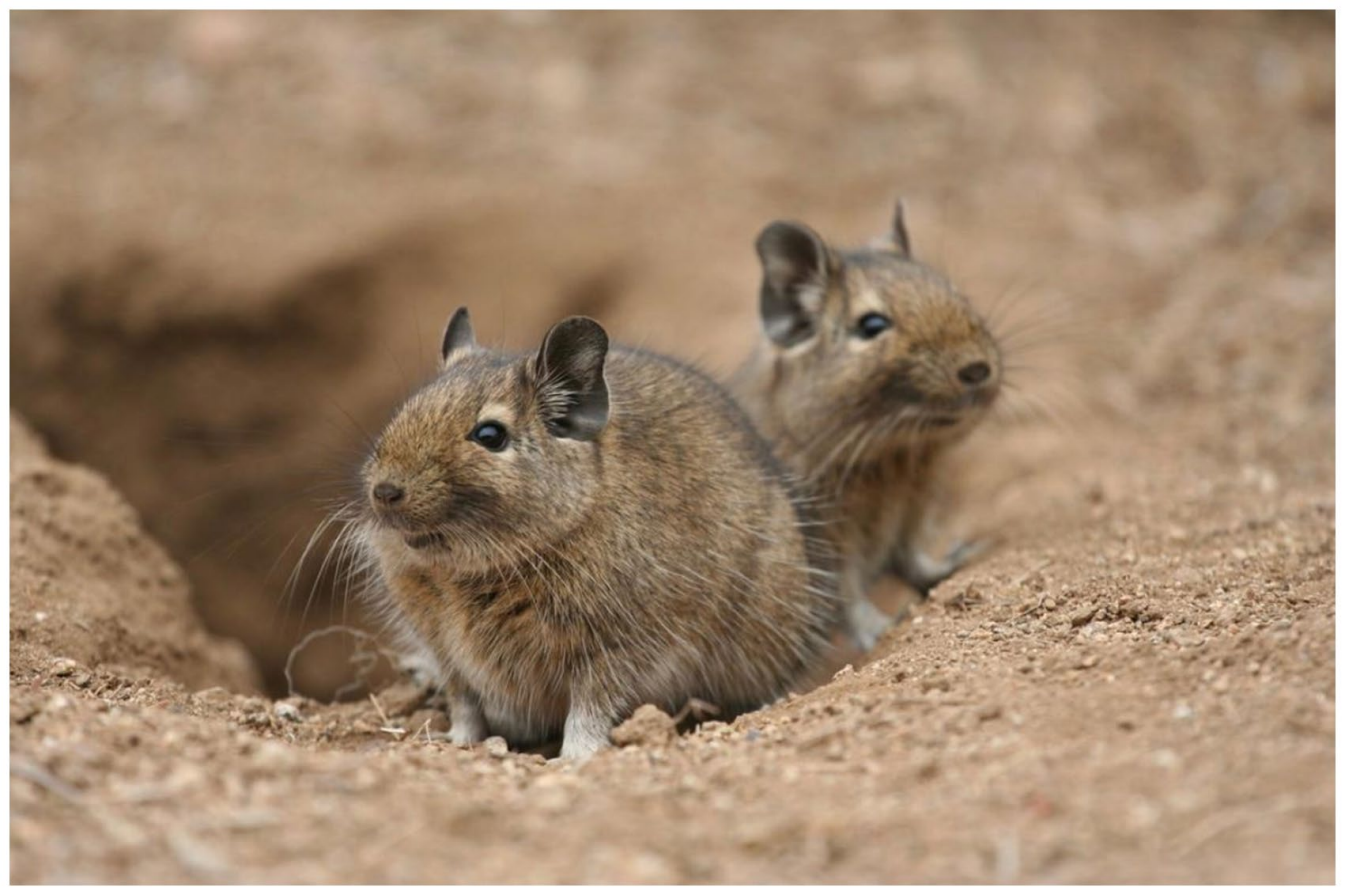
Two degus at their burrow entrance in the wild.

Although degus are mostly diurnal (active during daylight), in hot weather (>30°C), they may become crepuscular (active at dawn and dusk) to avoid the midday heat, retreating to their extensive burrow systems (Figure 3). Burrows may be dug communally, where animals form digging chains, similar to the naked mole rat (*Heterocephalus glaber*) of East Africa (Ebensperger and Bozinovic, 2000; Griffin, 2008) which are related to degus (Hurley et al., 2022). Burrow entrances are often marked by objects, such as small branches and feces, like the burrows of the pack rats (*Neotoma* species) of North America.

#### 2.1.2 Breeding in the wild

Degus are seasonal breeders, with mating usually occurring in May–June and lactation in September–October (Ebensperger and Hurtado, 2005; Quirici et al., 2010). Degus exhibit a relatively long gestation period of 90–95 days (Colby et al., 2012) with litters ranging from 1–12 pups (average of 6 ± 1) (Long and Ebensperger, 2010). Most males and females produce offspring with multiple partners, both from within their local and neighboring degu groups, the latter thought to decrease the risks associated with inbreeding (Ebensperger et al., 2019). Predation and seasonal breeding limits females to typically one breeding event during their entire lifetime, essentially making the degu an ecologically semelparous species (Ebensperger et al., 2013).

#### 2.1.3 Natural social behaviors

Degus live in small colonies with a strong social organization based on group territoriality. The burrow is the center of the defended territory. They typically live in groups of 2–10 individuals (mean ~3), with 0–3 males and 1–9 females (Ebensperger et al., 2016). Degu social units are rarely composed of only-male groups, while only-female units are commonly seen in addition to the more predominant multi-female and male-female groupings. Dominance hierarchies exist, with the dominant male and female having breeding rights (Ebensperger et al., 2004). However, social interactions extend beyond dominance; cooperation and altruism are also observed. Females of the same social group often rear their young in a common burrow (Ebensperger et al., 2006a, 2006b). They exhibit cooperative breeding, where females nurse not only their own young but also those of other females in the group (allonursing) (Ebensperger et al., 2006b). Sometimes even pups from a different species (Bennett’s chinchilla rat, *Abrocoma bennettii*, which sometimes lives alongside degus) are nursed, an unusual behavior in mammals (Mota-Rojas et al., 2021).

Degus have a surprisingly rich and complex repertoire of vocalizations used for various social interactions and expressing emotions that include whistles, chirps, moans, growls, grunts, and squeals. Their vocalizations include 15 distinct categories that vary with behavioral context and include ultrasonic and high-pitched vocalizations used primarily by pups to communicate with their mother and littermates. Short vocalizations are used in various contexts, like greeting, exploration, and mild annoyances. Low-pitched vocalizations express discontent, fear, or submission. Deep, threatening vocalizations are used in defensive situations or during disputes. Short, harsh sounds convey warning or threat, often preceding a more aggressive action. Loud, piercing calls are typically used during fights or intense fear (Long, 2007).

### 2.2 Ethological considerations of laboratory colony management, husbandry, and animal handling

#### 2.2.1 IACUC approvals and ethical considerations for establishing a colony

All research conducted on degus should be approved and comply with the ethical norms and regulations dictated by the relevant local, state, and federal entities of the host country. As our degu research is composed of an international collaboration between labs in Chile and the U.S., we have navigated the requirements for both countries. In Chile, our methods and procedures comply with the standards and regulations from the ethics committee of the Faculty of Sciences of the University of Chile and the Servicio Agrícola y Ganadero (SAG), as well as recommendations from the Animal Behavior Society of Chile. In the U.S., our work complies with the Animal Welfare Act, the DHHS “Guide for the Care and Use of Laboratory Animals”, the Institutional Animal Care and Use Committee (IACUC), and recommendations from the American Veterinary Medical Association.

#### 2.2.2 Degu trapping and capture

Researchers interested in or currently conducting research on degus should pay close attention to their animal’s origin, lineage, and genetic background. As degus are endemic to Chile, degu colonies in the U.S., Europe, and other areas around the world exhibit reduced genomic diversity due to inbreeding and limited opportunities to introduce genetically diverse individuals due to geographical limitations and international animal shipping restrictions. Previous studies using degus from different genetic backgrounds yielded differing results in regards to brain neuropathology (Inestrosa et al., 2005; Deacon et al., 2015; Steffen et al., 2016; Bourdenx et al., 2017; Tan et al., 2022). These features prompt caution. Degus from a genetically diverse population may not develop AD-like profiles in a uniform fashion, just as genetically diverse humans do not all develop AD.

Our studies have used outbred degus stocked from wild-caught animals originating from the Maipu region of Chile. The capture and transfer of degus from the wild to the laboratory colony settings should always be conducted by researchers trained in proper degu handling and preferably with prior experience handling animals that originate directly from the wild. Once an area likely to be inhabited by degus has been selected, an initial survey of the region should be conducted to locate burrow entrances and confirm their presence through fresh feces droppings or direct visual identification.

Sherman traps (25 cm × 8 cm × 10 cm; *L* × *W* × *H*) should be installed near burrow entrances, preferably those where degus or fresh feces were previously seen, before dawn. Oats and a few drops of vanilla extract are used to lure degus to the traps. Traps should be checked for capture every 30 min. During high temperature days this interval should be shortened (~every 15 min) to ensure animals are not subject to extended periods of high temperature. Once a degu has been captured, they are removed from the trap using a PVC tube (8 cm diameter). The degu, attempting to escape the trap, will enter the PVC tube, which can then be used to transfer them to an intermediary cage or container. We use a plastic box with wood shavings that is covered by a fitted metal grating lid.

Animals are then transferred to an initial holding room where they are weighed and their total body length, anus-to-snout, and right tarsus length are measured. Animals are then ear-tagged (or other identification methods are used, as described in the *animal identification* section below) with a unique ID number. Newly caught degus are transferred to the colony where they are grouped in same-sex metal cages (up to 5 animals) and observed for signs of aggression (if this happens, aggressive animals are separated into individual cages). Finally, animals are quarantined in a separate room where they are periodically inspected prior to full incorporation into the laboratory colony.

Captured pregnant females follow a similar protocol, with the only difference that after giving birth and waiting 3 months for pup weaning, they are returned to the wild. Reintroduction to the wild occurs at the same location they were captured to ensure they are close to their home burrow system. These dams retain their unique ID so that in the event of recapture, we can identify any related offspring in the degu colony.

#### 2.2.3 Laboratory colony space, cages and housing

**Figure 4:**
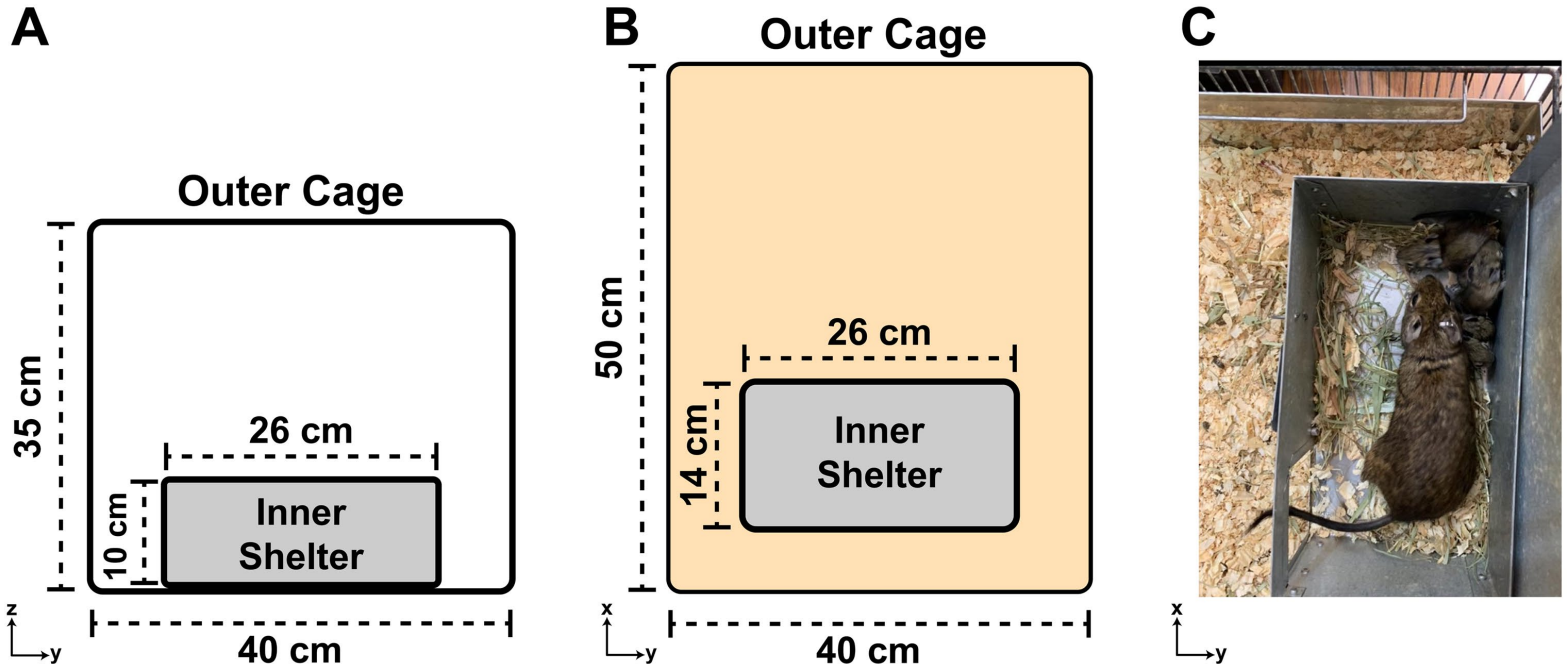
The degu cage design. **(A)** Diagram of a degu cage’s side view showing the width and height dimensions of the outer cage and inner shelter. **(B)** Same as **(A)**, but an overhead view of the cage with length and width dimensions noted. **(C)** Overhead view of an adult degu and her pups huddling inside the cage’s inner shelter. Note the inner shelter’s roof was lifted up for this picture, but should be kept shut for the degus.

Degus should be kept in a very strong wire mesh or barred cage (due to their proclivity to gnaw their surroundings) in a well-ventilated room, ideally around 20–22°C. They should never be housed in temperatures >30°C. In most laboratory rooms, a 12:12 h light:dark schedule is used. Solid-walled enclosures such as glass terrarium/aquariums are not suitable for degus housing due to poor ventilation. Large cages should be used; we use 50 cm × 40 cm × 35 cm (*L* × *W* × *H*) metal cages for up to 5 animals. We provide 26 cm × 14 cm × 10 cm metal boxes in the cages as inner “shelters” so that the degus can hide inside (Figure 4). They shelter under rocks and shrubs in the wild to avoid detection by aerial predators. Since degus live in groups in the wild, degus should never be housed individually (unless highly aggressive).

The cage base should be solid to prevent pododermatitis (bumblefoot, sore hocks) and other foot injuries. Gaps between bars, or mesh size, should be no more than 12 mm for adult degus (≥1 year). Standard 12 mm galvanized wire mesh, as typically used for rabbit hutches, is liable to be gnawed through, resulting in escape. Degus are one of the strongest and most persistent of gnawing rodents; the authors have witnessed large holes made in 2 mm thick steel plates. Bedding should always be provided. Straw and hay are ideal nest building materials that provide coverage for degus to hide in and are also part of their diet. Shredded paper, wood shavings, or sawdust should be dust-free, and never of resinous softwoods such as conifers, as these predispose degus to respiratory ailments. At least one chunk of hardwood or a wooden “toy” needs to be provided for them to gnaw on, which keeps their incisors free from excessive growth.

Cages should be changed and cleaned weekly during the day (Colby et al., 2012). When cleaning degu cages a small amount of old bedding, ~10%, should be mixed in with the new bedding so that a familiar cage odor is retained. This will minimize the risk of cage-changing induced stress and fighting in group- or pair-housed animals. Like many other mammals, male degus will also mark their territory with urine. Degus like to hoard food, so any food pieces that are going moldy in the bedding should be removed.

When adding new animals to an existing group, this is best done gradually. The first step involves moving the two cages closer together to allow visual/auditory/olfactory communication but not actual physical contact, then finally allow them to meet in neutral territory, like an entirely new cage. As with other rodents, introduction of a novel female can usually be accomplished without any fighting, while males, especially sexually mature ones, can prove much more difficult. It is best to attempt to incorporate co-caged animals with siblings, although success is not guaranteed (Colby et al., 2012).

#### 2.2.4 Laboratory colony maintenance and breeding

Degus can be kept in same-sex groups, or one male with one or two females in breeder cages. Females typically can reproduce for 4–4.5 years, while males can breed throughout their life. Reproductive maturity can be identified through penile spike development in males (2.5–3.5 months) and vaginal openings in females (3–3.5 months) (Hummer et al., 2007). However for optimal results, degus should be paired for breeding when they reach their adult body weight, which occurs around 6 months old (Colby et al., 2012). Although not a guarantee, pairing degus that have been able to interact for a few days before estrus increases the chances of successful breeding. Placing breeders in opaque cages can also reduce degu hyper-vigilance and, in turn, increase breeding chances (Palacios and Lee, 2013). It should be noted that alterations to the light:dark cycle (such as constant darkness, light, and dim red light) do not halt female estrous cycles.

Dystocia has been reported in degus and is most commonly seen in first pregnancies where there are a small number of very large pups that complicate the birth (Jekl et al., 2011b). Degus are precocial, meaning they are born completely furred, with eyes open, and able to walk in a coordinated manner (Edwards, 2009). There is no need to remove the male breeder after parturition as they do not show aggression towards newborns and, in some cases, immediate postpartum mating can occur. Furthermore, degu pups receive care from several adult degus in the wild that include maternal, paternal, and other group member interactions. As a highly social mammal, the amount of care and social interaction during the first few postnatal weeks are crucial for proper degu pup development (Bauer et al., 2016, 2019).

Although previous studies state that degu pups should be weaned at 4–6 weeks (Edwards, 2009; Colby et al., 2012; Palacios and Lee, 2013), we recommend weaning at 3 months (Jekl et al., 2011a) to enhance their developmental maturity in lab settings, always grouping them with at least one other degu. Females do not mate until after their young have been weaned (unless immediate postpartum copulation occurs) (Lee, 2004). If unwanted breeding is to be prevented, neutering of males is a viable option (Capello, 2005). Neutered males may live more peacefully in groups than un-neutered, but the latter will generally present few aggression-associated problems.

#### 2.2.5 Animal identification

Laboratory studies using group-housed animals are facilitated by easily identifying individual animals. The numerals on ear tags generally require careful restraint to be accurately read, and animal restraint immediately prior to a behavioral task such as maze running may cause stress and compromise behavioral performance. Even ear notches can be difficult to read. Another ear tag disadvantage is that animals sometimes scratch at them and tear them off or entangle their toes or claws in them.

Useful alternative ID techniques include coat markings or microchipping. The latter is an excellent permanent marker, although relatively expensive. Coat marking is inexpensive; using either a hydrogen peroxide solution, or preferably a human hair dye (blond ones work well) which is less irritating or toxic. Either should be applied to the surface of the fur in a discrete spot. Dip a cotton bud in the liquid, remove any excess, then contact the fur lightly and rotate the bud on its longitudinal axis to work the dye into the top layer of the coat, always twisting the bud in the direction of the fur. Alternatively, remove the hair from a small spot, using sharp scissors. Pinch a small tuft of hair between the thumb and index finger and keep the scissors firmly against the fingers to avoid cutting the skin of the animal. A good system that marks up to 9 animals is: one spot on left shoulder = 1, one spot right shoulder = 2, left flank = 3, right flank = 4, middle of back = 5, 5 +1 = 6, 5 + 2 = 7, etc. This system makes successive selection of animals for a behavioral task simple. To avoid testing the same animal twice, they can be placed temporarily in a holding cage. We find that the last animals to be removed from a group home cage are liable to have high levels of anxiety and tend to be difficult to catch, adding unwanted variability to the experiment.

Ear notching is best done under light anesthesia, for both ethical and practical reasons. Specialized punches are commercially available. Sterilize the punch tool before use and between animals. The system used in Experimental Psychology, Oxford (United Kingdom) was: one notch on the ventral part of the right ear = 1, two punches there, separated by ~3 mm, = 2, a notch on ear tip = 3, one on dorsal area = 5. Likewise, on the left ear, 10, 20, 30, 50. Thus 99 animals can be individually identified. Ear notching, in addition to the ideal microchipping method, is a viable way of marking newborn/juvenile degus if immediate identification is required.

Tail marking of degus, using a permanent marker pen, should be done very carefully, not only because of the risk of damage or even degloving, but also as it can stress the animal if the pressure is not as light as possible. The tail should be supported by a finger placed directly underneath the tail. A useful system is a dot = 1, a dash = 3. Examples of tail marking and ear notching can be seen in Deacon (2006b). Degus are not sexually dimorphic in physical appearance, so female/male identification is not possible without restraint and genitalia inspection.

#### 2.2.6 Considerations of laboratory enrichment settings

Degus are intelligent and playful, requiring various enrichment activities such as chewing toys, digging boxes, climbing structures, and foraging opportunities. Elevated platforms, hardwood tree branches and walkways, tunnels (e.g., PVC pipes as used for rainwater downpipes, at least 6 cm diameter, 15 cm + long), ramps, and ladders all provide enrichment and opportunities for exercise. Their positions should be changed occasionally to provide variety. A running wheel, preferably with a solid surface to avoid the risk of foot injury, is an almost mandatory provision, providing entertainment and exercise, which will minimize the risk of obesity. Rotate toys regularly to prevent boredom. It is good to offer them a “dust bath” of sand 2–3 times per week for 20 min. Ensure the baths are as free of urine and feces contamination as possible, although in the wild degu colony dust baths are marked with urine and anal gland secretions, so some soiling is inevitable.

#### 2.2.7 Laboratory diet

Water and food should be available *ad libitum*. Degus are adapted to the semi-arid environment of central and northern Chile, so their water intake is low, and the urine concentrated. Water should always be contained in a bottle with a drinking spout. The spout tip should be well above the cage floor (but within easy degu reach; longer spouts are usually required during juvenile life stages) to prevent leakage and wet bedding. Change the water, also clean the bottle and spout, at least every 3 days. Providing water in a bowl is not satisfactory; it will be full of bedding within hours if not minutes.

Hay should always be available, preferably the Timothy variety, which is especially palatable and often readily available from pet stores and laboratory suppliers. Avoid alfalfa hay as this leguminous plant has too much protein. Caviomorph pelleted food complements hay but should be given sparingly to minimize the risk of obesity. Prepared diets containing sugars such as molasses are to be rigorously avoided as degus are vulnerable to develop diabetes. Root vegetables may be given in small amounts (to limit sugar intake), also fresh greens such as grasses and dandelion leaves. Not only sugar content, but glycemic index (the rate at which sugar accumulates in the bloodstream), should be taken into consideration. Thus, although raw sweet potato has more sugar than white potatoes, its GI is much lower and therefore preferable. In captivity, degus can be fed with commercially available rabbit pellets (we use LabDiet 5321). Wheat-based diets, such as ProLab RMH 2000, should be used during both gestational and juvenile life stages to support growth. Some labs have reported pseudomonas infections in very young degus. This has not been our case, but if such conditions arise, water acidification during the first 3 months of age can help resolve the issue (Palacios and Lee, 2013).

#### 2.2.8 Potential health issues for laboratory degus

Dental problems are common in degus (especially in those over 2 year olds) [Jekl and Diplomate ECZM (Small Mammal), 2021]. Like all rodents, the incisors are elodont (continuously growing). A diet rich in hard vegetable matter, such as stems and bark, will check this growth, as will pieces of hardwood for gnawing (never softwood from conifers) placed in the cages. Overgrown incisor teeth should be clipped by experienced personnel or a veterinarian using a small pair of nail clippers or wire cutters. Paradoxically (to humans) white teeth are a sign of poor degu dental health. The natural color of degu incisors is orange yellow.

**Figure 5:**
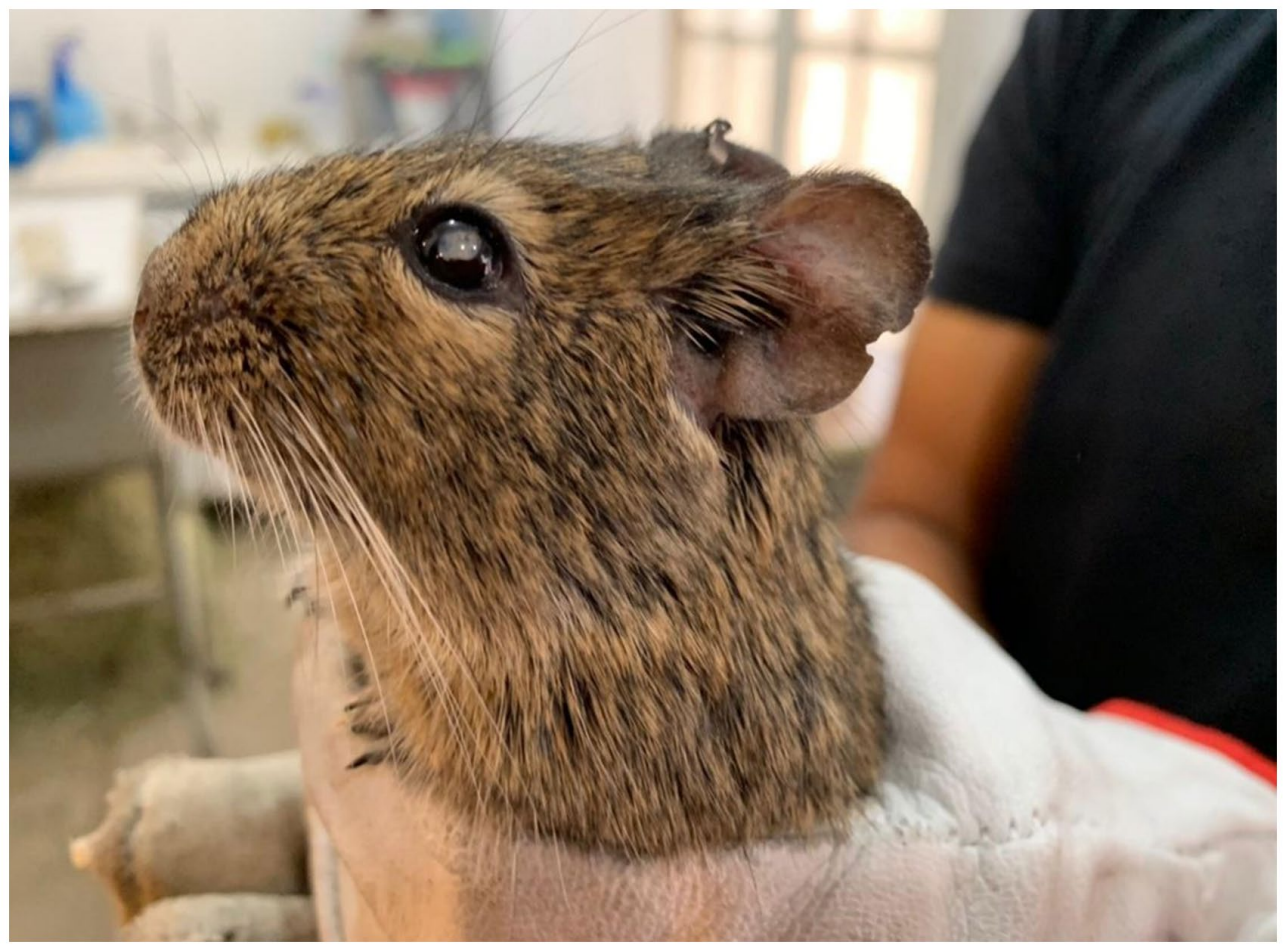
A degu with a cataract.

Degu insulin has a variant structure, thought to possess 10% or less of the activity of normal mammalian insulin, and is unable to efficiently regulate glucose levels. Although other compensatory mechanisms appear to be present to help control glucose levels (Opazo et al., 2004), this renders degus highly sensitive to high sugar diets and vulnerable to type 2 diabetes development. The condition typically manifests in cataract formation, fatty liver disease, and Langerhan islet hyperplasia in the degu (Colby et al., 2012; Chang et al., 2021) (a degu with cataracts can be seen in Figure 5). Researchers must keep this in mind when selecting a diet, habituation treats, and behavioral rewards.

In addition to diabetes, degus can develop atherosclerosis, cancer, infections, respiratory diseases, and several forms of body lesions. Degus fed a high fat and cholesterol diet have been shown to develop aortic atherosclerotic lesions along with increased cholesterol plasma levels (Homan et al., 2010). Although data on degu neoplasia is sparse, there have a been a few reports pointing towards a low tumor prevalence that seems to increase in older animals (Jekl et al., 2011b; Švara et al., 2020). Regular visual inspection, along with body weight recordings (the clearest indicator of an animal’s general health), are a must for identification of these and other conditions.

If degus persistently scratch their ears, a mite infestation may be present. There should be no body lesions, especially on the feet. Pododermatitis—inflamed calloused ventral surfaces—results from wire mesh floors, or dirty and wet solid ones. Respiration should be regular with no sounds of obstruction; the external nares clean and dry, without any signs of discharge. The anogenital area should be clean, with no signs of wetness or adhering fecal matter. Since in males the testes are within the abdomen, there is no obvious scrotum, unlike in mice or rats. The animal’s coat should be in a good glossy condition—an ungroomed dull coat betrays illness, such as diabetes. Barbering is common in degus, especially in those younger than 2 years after being triggered by some stressful event, such as a small cage size, insufficient exercise, or a lack of burrowing material [Jekl and Diplomate ECZM (Small Mammal), 2021]. An alert, active animal that responds to the observer is normally in good health.

#### 2.2.9 Anesthesia and euthanasia

If it is necessary to anaesthetize a degu, sevoflurane or isoflurane work well. Injectable anesthetics include ketamine combinations (Colby et al., 2012) and zoletil (a combination of tiletamine and zolazepam) with xylazine. The latter mixture is recommended as it has minimal depressant effects on respiratory/cardiovascular function and a high therapeutic index. However, it is not so instantly controllable as inhalation anesthetics. Avoid potentially hepatotoxic medications such as halothane, also methoxyflurane, as degus are susceptible to liver disease (Colby et al., 2012; Bament, 2013). Degus have a high tolerance to morphine, making it an unsuitable analgesic. Carprofen is a suitable non-opioid analgesic. Colby et al. (2012) lists suitable analgesics and anesthetics along with doses.

If a degu must be euthanized, there are several different techniques. The experimenter should be tutored on these to gain technical proficiency.

- Lethal injection: Administer a large overdose of an anesthetic such as euthatal/euthasol (pentobarbitone, 150–200 mg/kg).
- Isoflurane: Place the animal(s) in a euthanasia chamber and administer isoflurane at a slow rate (1%) using a vaporizer and increasing to 5% when the animal loses consciousness. The rate of administration will then be increased until the animal stops breathing.
- Carbon dioxide: Place the animal(s) in a euthanasia chamber and slowly increase the CO_2_ concentration until all respiration has ceased. Leave for 10–30 min to confirm death by rigor mortis.
- Confirmation of death: Rigor mortis or prolonged cessation of respiration followed by decapitation.

### 2.3 Degu behavioral methods

Our laboratory and others have demonstrated degus are able to perform memory tasks and follow traditional protocols for memory evaluation, as well as combined protocols that assess both working and reference memory. Behavior tests such as open field, novel object recognition, object location, delayed T-maze, radial arm maze, Barnes maze, light-dark test, social isolation, maternal behaviors, and tool-use, among others, have been successfully employed in previous degu studies (Okanoya et al., 2008; Popović et al., 2009, 2010; Colonnello et al., 2011; Ardiles et al., 2012; Kumazawa-Manita et al., 2013; Bauer et al., 2016; Valdivia et al., 2023). Researchers intending to set up a degu colony with behavioral testing should be prepared to reconfigure their behavior rooms and protocols to accommodate degus. Here we report detailed procedures and methodical information for performing the burrowing behavior test in degus.

#### 2.3.1 Behavior testing room requirements

In addition to the space necessary for the degu colony, at least 3 additional rooms are required for behavioral testing: one for habituation, another for behavior experiments, and a final one for test observation and recording. All these rooms require temperature, humidity, and light-cycle control capabilities, all of which should match the colony settings. Additional space, ideally close to the behavior room, is required to store the behavior test components (e.g., mazes, tubes, objects), which are considerably large for degus.

Behavior rooms (at least 4 m × 4 m) should be equipped with a camera mounted on the room ceiling or a modular rig to record behavior tasks. The adjacent observation room (at least 2 m × 3 m) should have a computer with behavior analysis software (e.g., Noldus EthoVision) that is connected to the camera. The habituation room (at least 4 m × 4 m) acts as an intermediary between the colony and the behavior room. Animals should be transported to the habituation room at least 30 min before behavior experiments are scheduled. This setup allows for efficient testing of large numbers of degus, where one researcher is tasked with transporting the degus between the habituation and behavior rooms, and another is responsible for recording the tasks in the observation room.

#### 2.3.2 Habituation to human handling

As with all species, the younger an animal is when first handled, the tamer it will become. Many researchers and pet owners, however, do not have this opportunity if the animals are acquired as adults, so a progressive habituation regime is required. This is essential to tame the animal and build a “working relationship.” Unlike laboratory or pet rats and mice, the degu does not have a long history of domestication, and wild-caught individuals can be extremely slow to tame.

The key to any animal training is patience and moving forward one step at a time (Deacon, 2006b). The animal needs to develop a positive attitude towards the trainer, and the best way to achieve this is to offer small food treats. Using a gloved hand, allow the degu(s) to approach, sniff it, and retrieve the treat. Small pieces of carrot, sweet potato etc. may work but always ensure that the degu has first been offered this in the home cage prior to the habituation session. All animals are slightly wary of novel foods (hyponeophagia or neophobia). Degus love sunflower seeds, but these should only be given in small amounts as they are very fattening. If they do not approach your hand, move on to the next cage, after rubbing some of the bedding material with your hands to allow them to become used to this novel odor. Subsequently, gradually approach the animal until you can touch and gently stroke it (always in the direction that the fur lies, i.e., rostral-caudal, head to tail). The flank or middle of the body is best; avoid touching near the head, as this is a more sensitive or reactive area. Gradually increase your touch to a firm pressure, sometimes massaging the body gently but firmly. Many short handling sessions work best, as the intervals allow high arousal/anxiety levels to subside. Although habituation times can vary from degu to degu (e.g., if animals originate from the wild), six short (1–2 min) handling sessions separated by 10–20 min intervals should produce a notable habituation effect, making the animals much easier to handle on future occasions. After extensive habituation, with lots of stroking, the degu may even reciprocate by “grooming” your hand or gently nibbling it. Reinforce habituation procedures by offering occasional treats.

Eventually you should be able to pick the degu up and support it close to your chest. Be sure to always provide support from underneath, or else it will likely struggle and try to escape. Never pick degus up by the tail, as, especially if held tightly, the skin may slip off (deglove). If this occurs, a veterinarian should be called to amputate the tail properly to avoid infection. Avoid forcefully cornering a degu unless this is absolutely essential to catch it. “Cupping” the degu to pick it up with both hands, moving one hand to cover the back and prevent escape, is the best capture technique. Always approach from the side, as aerial raptors characteristically swoop down on their prey from above, and to the degu, a human is perceived as a potentially threatening predator. Owls and eagles are the most common predators of degus in the wild, although some degus are taken by foxes.

The ultimate goal of habituation to human handling in the laboratory is to make the animals more amenable to experimental procedures that yield reproducible results, in particular the handling required to transfer the animal from its cage to the experimental situation, e.g., a maze. Alternatively, experimenters may prefer to transfer animals by a tunnel (e.g., PVC tube), rather than using their hands. This virtually eliminates the possibility of the experimenter being bitten and reduces the incidence of degu escape. Tunnel transferring has been shown to cause markedly less stress than manual handling in mice (Sensini et al., 2020). The best practice for tunnel transfer is to put the tunnel along one wall of the cage and allow the animal to voluntarily enter it, perhaps with gentle encouragement from the hands of the experimenter behind the degu, without actually touching the animal. We recommend never chasing the animal around the cage with the tunnel. Tunnels open at both ends are best, or ones with removable caps, as a degu may choose to stay in the closed dark end of a capped tunnel. Gently blowing down the tunnel can encourage the degu to exit the tunnel.

### 2.4 *Apoe* genotyping

**Figure 6:**
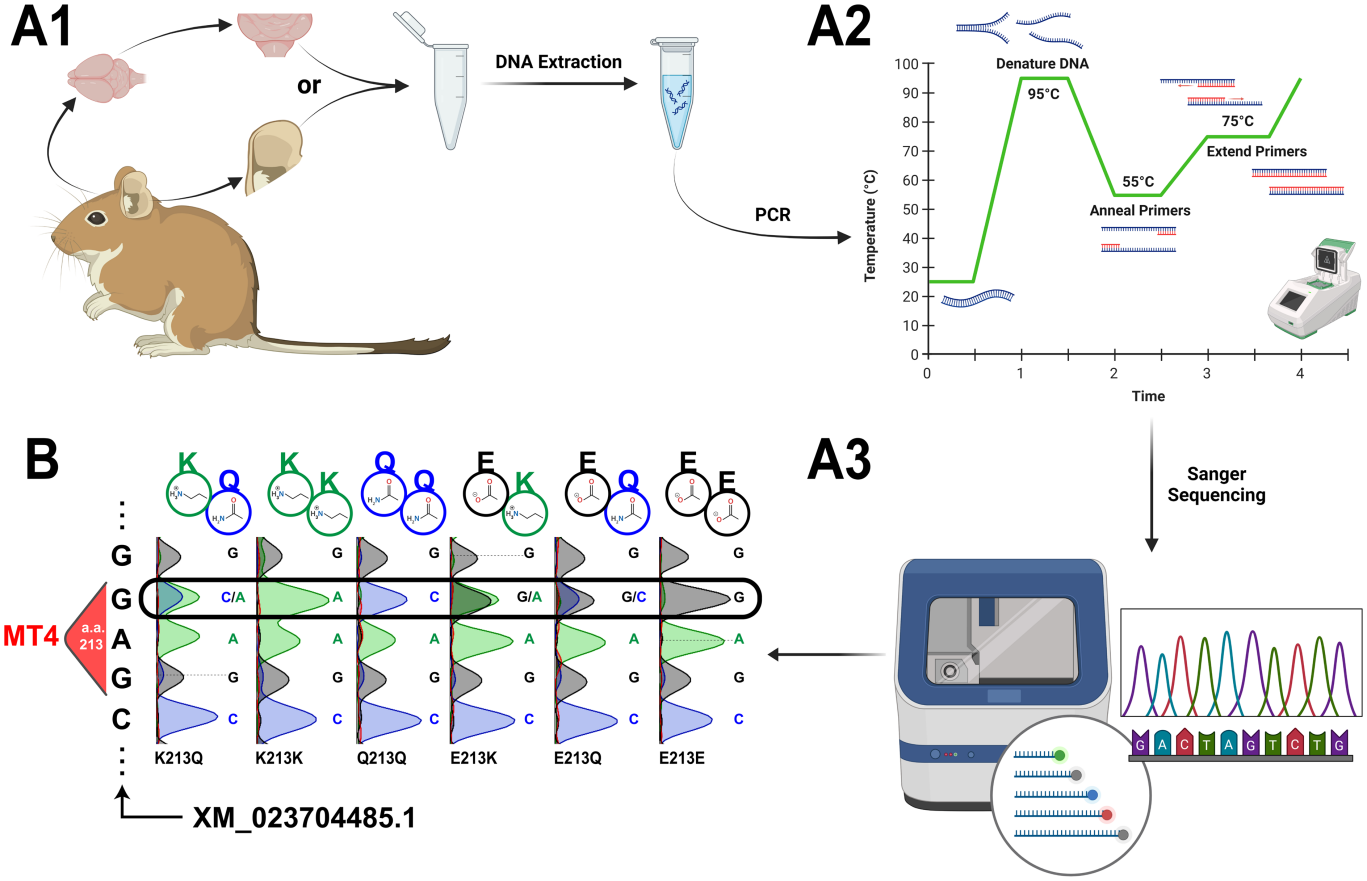
*Apoe* Mt4 (amino acid 213) genotyping in the degu. **(A)** Experimental outline for degu *Apoe* Mt4 genotyping. **(A1)** Experiment begins with tissue extraction of a small amount of frozen brain (at least 25 mg) or ear tissue (preferred over tail clippings) that is followed by DNA extraction. **(A2)** The *Apoe* Mt4-containing amplicon is then amplified from the purified DNA via PCR and sent for **(A3)** Sanger sequencing. **(B)** Representative traces from all possible degu *Apoe* Mt4 genotypes using our protocol. Sanger sequencing results were aligned to Mt4 and the degu *Apoe* reference (XM_023704485.1) using the MUSCLE algorithm. The resulting amino acid residues for each genotype are denoted above each sequencing trace. Created in BioRender (https://BioRender.com/p88v181).

We have an established protocol for degu *Apoe* Mt4 genotyping via PCR amplification followed by Sanger sequencing. Following DNA extraction from degu ear or flash frozen brain tissue (at least 25 mg), purified DNA (20–40 ng) is added to a master mix with the degu *Apoe* primers (final total volume of 25 μL) (Table 1 and Figure 6A1). PCR initial denaturation runs for 2 min at 95°C, is followed by 35 cycles of 30 s of denaturation at 95°C, 30 s of annealing at 55°C, elongation for 40 s at 72°C (Figure 6A2), and finished with 5 min of elongation at 72°C. The resulting 631 bp amplicon is then sent for Sanger sequencing (Figure 6A3). Results are aligned to the degu *Apoe* reference (XM_023704485.1) and a 9 bp sequence containing Mt4 and its flanking codons (CAG**GAG**CGC) using the MUSCLE algorithm (Edgar, 2004) to determine the animal’s genotype (Figure 6B).

**Table 1:** Degu *Apoe* Mt4 genotyping primers.

Gene SNP	Forward primer	Reverse primer
*Apoe* Mt4	5′-GGTGCTCATGGAAGACACCA-3′	5′-CTTCTCGATGAGATCGGCCC-3′

Forward and reverse primers flanking the Mt4 SNP site on the degu *Apoe* gene. PCR amplification using these primers can be followed by Sanger sequencing and alignment to the degu *Apoe* reference sequence (XM_023704485.1) to identify degu *Apoe* Mt4 genotypes.

## 3 Results

### 3.1 Burrowing behavior testing in the degu

**Figure 7:**
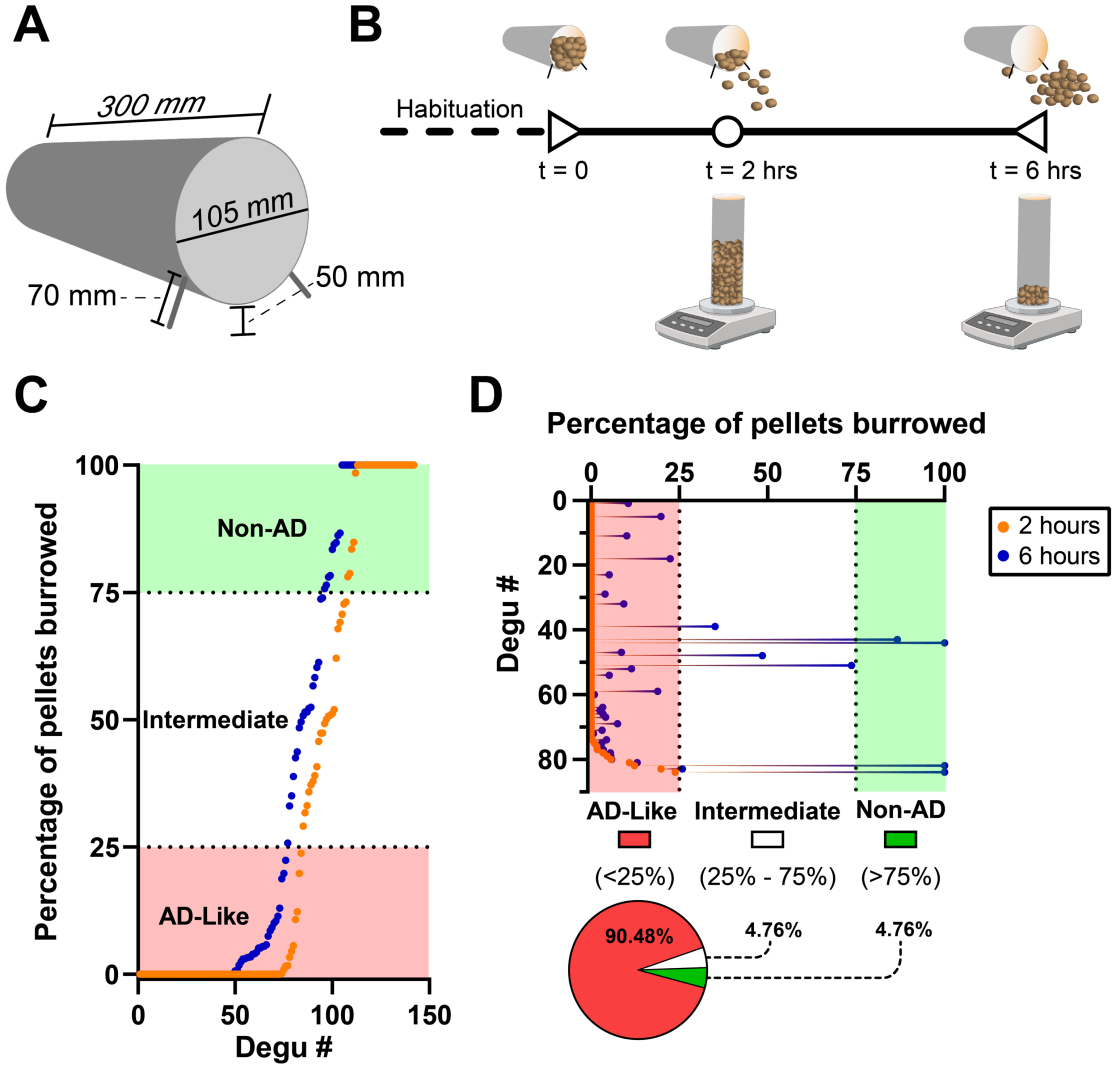
The burrowing behavior test in a cohort of 142 4–5.5-year-old degus. **(A)** Dimensions of a burrowing tube. **(B)** Burrowing behavior experiment timeline denoting the time points (2 and 6 total hours) at which the grams of remaining (not burrowed) pellets should be weighed. Note this should be repeated 2 more times (total of 3 excavation tests) with 48 h between tests to improve degu burrowing ability and reduce variability. **(C)** Plot of ranked burrowing performance after 2 (orange data points) and 6 h (blue data points). Degus are classified as AD-like if they burrow <25% of the pellets and non-AD if they burrow >75%. **(D)** Details of the ranked 2-h data from the first 84 degus of plot **(C)** that were classified as AD-like. Performance at the 6-h mark shows four (4.76%) of these degus improve to intermediate levels and another four (4.76%) reach Non-AD performance levels. Created in BioRender (https://BioRender.com/z37y001).

Our group’s studies demonstrated the burrowing behavior paradigm is capable of identifying a subpopulation of degus with species-typical burrowing deficits that correlate with the presence of AD-like neuropathologies (Tan et al., 2022). This test is inexpensive, relatively simple to set up, and highly sensitive to several types of brain diseases/conditions (Deacon, 2006a). A plastic tube (300 mm long, 105 mm in diameter) is raised 5 cm on one end by screwing two 7 cm screws (Figure 7A). The opposite end is capped, and the tube is filled with 1,400 grams of pellets. The tube with pellets is then placed in a degu cage and is ready for testing (Deacon, 2006a, 2009; Deacon et al., 2015).

Prior to burrowing testing, degus should undergo handling habituation to reduce the amount of stress they undergo when transferred from their home cage to the burrowing behavior cage. A burrowing tube (without pellets) can also be placed in their home cage to familiarize them with the apparatus, although researchers should be wary that most degus will damage the tubes by gnawing on them. Following habituation, degus are placed in the cage and allowed to burrow for 2 h. Burrowing involves the degu removing material from the burrowing tube. After this, the burrowing tube is retrieved from the cage, weighed, and returned back to the cage. The same is done 4 h after (total of 6 burrowing hours), yielding the amount of weight burrowed after 2 and 6 h (Figure 7B). The entire test is repeated two more times (tests should be 48 h apart) to improve burrowing ability and reduce variability (Deacon, 2006a). Using the last (3rd) burrowing test run, our group classifies degus as AD-like if they burrow less than 25% (350 grams) of the pellets and Non-AD degus if they burrow more than 75% (1,050 grams) (Tan et al., 2022) (Figure 7C). Either of the 2- or 6-h measurement timepoints may be used for classification depending on the study’s focus and design. Although the 2-h mark is less affected from the ceiling effect seen at the 6-h mark, researchers should keep in mind that some animals, even those that perform very poorly after 2-h, can dramatically increase their burrowing activity after a few more hours (Figure 7D).

The burrowing data in this study was replotted and reanalyzed from Tan et al. (2022). All these degus came from a genetically diverse degu colony at the Institute of Ecology and Biodiversity, University of Chile, Santiago, Chile, that followed all of the colony, laboratory, and experimental principles mentioned in the methods section.

### 3.2 Burrowing behavior performance and test duration comparison in an aged degu population

Burrowing behavior tests from a cohort of 142 degus (replotted from Tan et al., 2022) shows more than half (53.5%) of the 4–5.5 year old outbred degu population exhibits AD-like burrowing performance (<25% of the burrowing pellets) at the 6-h mark (Figure 7C). Around a third (33.1%) of them maintain healthy Non-AD burrowing levels (>75% of pellets), and a small group of them (13.4%) burrow between 25–75% of the burrowing pellets, which we call “intermediate” burrowers (Figure 7C).

We further analyzed this dataset to determine if there were any considerable burrowing improvements between the 2-h and 6-h marks. Focusing on the degus that were classified as AD-like after 2 h of burrowing, we find that a small percentage of these animals migrate to the intermediate (4 degus, 4.76%) and Non-AD (4 degus, 4.76%) levels after 4 additional burrowing hours (Figure 7D). These findings show degus, even those showing AD-like burrowing levels after 2 h, are capable of substantial improvements if given more time in the burrowing cage.

### 3.3 Degu *Apoe* Mt4 genotyping via PCR and sanger sequencing

Our group identified a correlation between *Apoe* Mt4 (amino acid 213) genotype and the manifestation of both burrowing deficits and AD-like pathology in the degu brain. The Mt4 SNP site can yield 3 different residues (glutamine, lysine, and glutamic acid), which is thought the affect the degu’s APOE interaction dynamics, similar to what is seen in humans, and contribute to the manifestation of pathological brain aggregates (Hurley et al., 2022).

Based on previous work, we expected to identify six possible *Apoe* Mt4 genotypes in the degu: E213E (43% prevalence in the outbred degu population), E213Q (31%), E213K (11%), Q213Q (13%), Q213K (1%), and K213K (1%) (Hurley et al., 2022). Following the protocol detailed here (Figure 6A), we are able to amplify an Mt4-containing segment of degu *Apoe* that was subsequently Sanger sequenced. We show representative traces for the six *Apoe* Mt4 (amino acid 213) genotypes present in degus after alignment to *Apoe* (XM_023704485.1) (Figure 6B).

## 4 Discussion

Degus have captured considerable attention as animal models for aging and AD. Although degu research in the U.S. is thought to have started in the 1960’s (McMurray, 2024), it was not until the 2000’s that AD-like brain pathologies were investigated in the degu (Inestrosa et al., 2005). The discovery of natural AD-like pathology in their brains gradually gained saliency, coinciding with the failure of most AD clinical trials and the projected increase in the number of AD patients in the coming decades (Alzheimer’s Association Report, 2024). Better animal models may yield better therapies (Polis and Samson, 2024). As much of the present basic research scaffolding is based on studies conducted on transgenic mice, this motivates the efforts to identify an organism that exhibits naturally occurring AD pathology and is amenable to laboratory settings at low costs.

The degu is one such model that can exhibit AD-like neuropathology and has a history as a successful laboratory animal model. A portion of the aged outbred degu population naturally exhibits a rich repertoire of neuropathological markers similar to those seen in patients with AD. Animals exhibiting these pathological aggregates correlatively manifest deficits in burrowing behavior, a species-typical task thought to reflect the activities of daily living AD patients struggle with (Deacon, 2019; Tan et al., 2022). These characteristics position the long-lived degu as a promising rodent model of neurodegeneration in aging and sporadic AD research.

Although the majority of degu research has not focused on AD, there are many earlier studies involving neuroscience and behavioral analysis. These earlier studies provide a scaffold of protocols and materials for degu AD research. As degus are rodents, many of the reagents, procedures, and experiments conducted on mice and rats can be successfully applied to degus. However, it is paramount to maintain an ethological perspective when it comes to transferring a mouse or rat protocol to degus. Simply calculating body size/weight ratios between the degu and the mouse/rat to design colony cages/apparatus or using established mouse behavior protocols would neglect intrinsic degu ethological considerations, such as their sociality, diurnality, and increased visual acuity. These features need to be considered for proper housing and experimentation.

The degu’s natural properties dictate their susceptibility to other diseases besides AD. In particular, the degu’s natural habitat imposes on them a rather restrictive diet in the wild. This makes degus susceptible to various metabolic-related diseases. Most prominent among these are type 2 diabetes and cardiovascular diseases, both of which are known AD comorbidities. This places degus as an attractive model for not only investigating each of these conditions individually, but also how they collectively contribute to each other’s pathogenesis and progression. Researchers should thus carefully monitor their animal’s diet and weight, as inadvertent metabolic disease could also be a confounding source during data interpretation.

Our article provides a comprehensive guide to degu housing and behavioral experimentation based on published literature and our research group’s professional experience. Standards and instructions for wild outbred degu trapping and assimilation into a colony setting are also included. As there seem to be neuropathological discrepancies in prior studies between outbred and lab inbred degu populations, colony managers should carefully arrange their breeder cages and consider refreshing their colony’s genetic pool with genetically diverse degus at regular intervals (if access to outbred degus is available). They should also consider incorporating periodic behavioral testing, such as our detailed burrowing paradigm, to gauge degu cognitive states and inform future neuropathological results.

We additionally provide our *Apoe* genotyping protocol and sample sequencing results for all degu *Apoe* genotype permutations, as this AD-related gene in humans also exhibits AD-like profiles in the degu in a genotype-dependent manner (Hurley et al., 2022; Zampieri et al., 2024). Further genetic characterization of the degu should help identify genetic markers that are relevant to AD and other diseases.

Lastly, there is extensive “non-professional” literature on degus, often produced by pet owners. While these references may contain interesting anecdotal material, most such references are not peer-reviewed, so caution should be applied before implementing some of their recommendations.

## Data Availability

The original contributions presented in the study are included in the article/supplementary material, further inquiries can be directed to the corresponding authors.

## References

[ref1] Akers K. G. Martinez-Canabal A. Restivo L. Yiu A. P. De Cristofaro A. Hsiang H. L.. (2014). Hippocampal neurogenesis regulates forgetting during adulthood and infancy. Science 344, 598–602. doi: 10.1126/science.1248903, PMID: 24812394

[ref2] Alzheimer’s Association Report (2024). 2024 Alzheimer’s disease facts and figures. Alzheimers Dement. 20, 3708–3821. doi: 10.1002/alz.13809, PMID: 38689398 PMC11095490

[ref3] Ardiles A. O. Ewer J. Acosta M. L. Kirkwood A. Martinez A. D. Ebensperger L. A.. (2013). *Octodon degus* (Molina 1782): a model in comparative biology and biomedicine. Cold Spring Harb. Protoc. 2013, 312–318. doi: 10.1101/pdb.emo071357, PMID: 23547147 PMC4386868

[ref4] Ardiles Á. O. Tapia-Rojas C. C. Mandal M. Alexandre F. Kirkwood A. Inestrosa N. C.. (2012). Postsynaptic dysfunction is associated with spatial and object recognition memory loss in a natural model of Alzheimer’s disease. Proc. Natl. Acad. Sci. U.S.A. 109, 13835–13840. doi: 10.1073/pnas.1201209109, PMID: 22869717 PMC3427050

[ref5] Bament W. (2013). A VN’s guide to degus part two: common health issues. Vet. Times. Available at: https://www.vettimes.co.uk/app/uploads/wp-post-to-pdf-enhanced-cache/1/a-vns-guide-to-degus-part-two-common-health-issues.pdf. (Accessed May 1, 2013)

[ref6] Bauer C. M. Correa L. A. Ebensperger L. A. Romero L. M. (2019). Stress, sleep, and sex: a review of endocrinological research in *Octodon degus*. Gen. Comp. Endocrinol. 273, 11–19. doi: 10.1016/j.ygcen.2018.03.014, PMID: 29545088

[ref7] Bauer C. M. Ebensperger L. A. León C. Ramírez-Estrada J. Hayes L. D. Romero L. M. (2016). Postnatal development of the degu (*Octodon degus*) endocrine stress response is affected by maternal care. J. Exp. Zool. A 325, 304–317. doi: 10.1002/jez.2018, PMID: 27198697

[ref8] Bourdenx M. Dovero S. Thiolat M.-L. Bezard E. Dehay B. (2017). Lack of spontaneous age-related brain pathology in *Octodon degus*: a reappraisal of the model. Sci. Rep. 7:45831. doi: 10.1038/srep45831, PMID: 28374864 PMC5379186

[ref9] Capello V. (2005). Prescrotal open technique for neutering a degu. Exotic DVM 6, 29–31.

[ref10] Chang L. Y.-L. Ardiles A. O. Tapia-Rojas C. Araya J. Inestrosa N. C. Palacios A. G.. (2020). Evidence of synaptic and neurochemical remodeling in the retina of aging degus. Front. Neurosci. 14:161. doi: 10.3389/fnins.2020.00161, PMID: 32256305 PMC7095275

[ref11] Chang L. Y.-L. Palanca-Castan N. Neira D. Palacios A. G. Acosta M. L. (2021). Ocular health of *Octodon degus* as a clinical marker for age-related and age-independent neurodegeneration. Front. Integr. Neurosci. 15:665467. doi: 10.3389/fnint.2021.665467, PMID: 33927598 PMC8076605

[ref12] Cisternas P. Zolezzi J. M. Lindsay C. Rivera D. S. Martinez A. Bozinovic F.. (2018). New insights into the spontaneous human Alzheimer’s disease-like model *Octodon degus*: unraveling amyloid-β peptide aggregation and age-related amyloid pathology. J. Alzheimers Dis. 66, 1145–1163. doi: 10.3233/JAD-180729, PMID: 30412496

[ref13] Cogram P. Garduño B. M. Ren B. Xu X. (2024). First international conference on unconventional animal models of Alzheimer’s disease and aging. J. Alzheimers Dis. 98, 333–336. doi: 10.3233/JAD-249004, PMID: 38393919 PMC11090612

[ref14] Colby L. A. Rush H. G. Mahoney M. M. Lee T. M. (2012). “Chapter 44—degu” in The laboratory rabbit, guinea pig, hamster, and other rodents. eds. Suckow M. A. Stevens K. A. Wilson R. P. (Boston, MA: Academic Press), 1031–1053.

[ref15] Colonnello V. Iacobucci P. Fuchs T. Newberry R. C. Panksepp J. (2011). *Octodon degus*. A useful animal model for social-affective neuroscience research: basic description of separation distress, social attachments and play. Neurosci. Biobehav. Rev. 35, 1854–1863. doi: 10.1016/j.neubiorev.2011.03.014, PMID: 21477615

[ref16] Contreras L. Torres-Mura J. Yáñez J. (1987). Biogeography of octodontid rodents: an eco-evolutionary hypothesis. Field. Zool. 39, 283–287.

[ref17] Deacon R. M. J. (2006a). Burrowing in rodents: a sensitive method for detecting behavioral dysfunction. Nat. Protoc. 1, 118–121. doi: 10.1038/nprot.2006.19, PMID: 17406222

[ref18] Deacon R. M. J. (2006b). Housing, husbandry and handling of rodents for behavioral experiments. Nat. Protoc. 1, 936–946. doi: 10.1038/nprot.2006.120, PMID: 17406327

[ref19] Deacon R. M. J. (2009). Burrowing: a sensitive behavioural assay, tested in five species of laboratory rodents. Behav. Brain Res. 200, 128–133. doi: 10.1016/j.bbr.2009.01.007, PMID: 19373978

[ref20] Deacon R. M. (2019). Dementia and animal activities of daily living. J. Psychiatry Cogn. Behav. 4:155. doi: 10.29011/2574-7762.000055

[ref21] Deacon R. M. J. Altimiras F. J. Bazan-Leon E. A. Pyarasani R. D. Nachtigall F. M. Santos L. S.. (2015). Natural AD-like neuropathology in *Octodon degus*: impaired burrowing and Neuroinflammation. Curr. Alzheimer Res. 12, 314–322. doi: 10.2174/1567205012666150324181652, PMID: 25817252

[ref22] Ebensperger L. A. Bozinovic F. (2000). Communal burrowing in the hystricognath rodent, *Octodon degus*: a benefit of sociality? Behav. Ecol. Sociobiol. 47, 365–369. doi: 10.1007/s002650050678

[ref23] Ebensperger L. A. Correa L. A. León C. Ramírez-Estrada J. Abades S. Villegas Á.. (2016). The modulating role of group stability on fitness effects of group size is different in females and males of a communally rearing rodent. J. Anim. Ecol. 85, 1502–1515. doi: 10.1111/1365-2656.12566 27365190

[ref24] Ebensperger L. A. Correa L. A. Ly Prieto Á. Pérez de Arce F. Abades S. Hayes L. D. (2019). Multiple mating is linked to social setting and benefits the males in a communally rearing mammal. Behav. Ecol. 30, 675–687. doi: 10.1093/beheco/arz003

[ref25] Ebensperger L. A. Hurtado M. J. (2005). Seasonal changes in the time budget of degus, *Octodon degus*. Behaviour 142, 91–112. doi: 10.1163/1568539053627703, PMID: 38220602

[ref26] Ebensperger L. A. Hurtado M. J. Ramos-Jiliberto R. (2006a). Vigilance and collective detection of predators in degus (*Octodon degus*). Ethology 112, 879–887. doi: 10.1111/j.1439-0310.2006.01242.x

[ref27] Ebensperger L. A. Hurtado M. J. Soto-Gamboa M. Lacey E. A. Chang A. T. (2004). Communal nesting and kinship in degus (*Octodon degus*). Naturwissenschaften 91, 391–395. doi: 10.1007/s00114-004-0545-5, PMID: 15309311

[ref28] Ebensperger L. A. Hurtado M. J. Valdivia I. (2006b). Lactating females do not discriminate between their own young and unrelated pups in the communally breeding rodent, *Octodon degus*. Ethology 112, 921–929. doi: 10.1111/j.1439-0310.2006.01251.x

[ref29] Ebensperger L. A. Sobrero R. Quirici V. Castro R. A. Tolhuysen L. O. Vargas F.. (2012). Ecological drivers of group living in two populations of the communally rearing rodent, *Octodon degus*. Behav. Ecol. Sociobiol. 66, 261–274. doi: 10.1007/s00265-011-1274-3, PMID: 22344477 PMC3277426

[ref30] Ebensperger L. A. Tapia D. Ramírez-Estrada J. León C. Soto-Gamboa M. Hayes L. D. (2013). Fecal cortisol levels predict breeding but not survival of females in the short-lived rodent, *Octodon degus*. Gen. Comp. Endocrinol. 186, 164–171. doi: 10.1016/j.ygcen.2013.02.044 23524002

[ref31] Edgar R. C. (2004). MUSCLE: multiple sequence alignment with high accuracy and high throughput. Nucleic Acids Res. 32, 1792–1797. doi: 10.1093/nar/gkh340, PMID: 15034147 PMC390337

[ref32] Edwards M. S. (2009). Nutrition and behavior of degus (*Octodon degus*). Vet. Clin. North Am. Exot. Anim. Pract. 12, 237–253. doi: 10.1016/j.cvex.2009.01.003, PMID: 19341951

[ref33] Garduño B. M. Hanni P. Hays C. Cogram P. Insel N. Xu X. (2024). How the forebrain transitions to adulthood: developmental plasticity markers in a long-lived rodent reveal region diversity and the uniqueness of adolescence. Front. Neurosci. 18:18. doi: 10.3389/fnins.2024.1365737 (PMID: 38456144 PMC10917993

[ref34] Griffin A. S. (2008). Naked mole-rat. Curr. Biol. 18, R844–R845. doi: 10.1016/j.cub.2008.07.054, PMID: 18812073

[ref35] Hagenauer M. H. Lee T. M. (2008). Circadian organization of the diurnal caviomorph rodent, *Octodon degus*. Biol. Rhythm. Res. 39, 269–289. doi: 10.1080/09291010701683425

[ref36] Homan R. Hanselman J. C. Bak-Mueller S. Washburn M. Lester P. Jensen H. E.. (2010). Atherosclerosis in *Octodon degus* (degu) as a model for human disease. Atherosclerosis 212, 48–54. doi: 10.1016/j.atherosclerosis.2010.06.004, PMID: 20630529

[ref37] Hummer D. L. Jechura T. J. Mahoney M. M. Lee T. M. (2007). Gonadal hormone effects on entrained and free-running circadian activity rhythms in the developing diurnal rodent *Octodon degus*. Am. J. Physiol. Regul. Integr. Comp. Physiol. 292, R586–R597. doi: 10.1152/ajpregu.00043.2006, PMID: 16917014

[ref38] Hurley M. J. Urra C. Garduno B. M. Bruno A. Kimbell A. Wilkinson B.. (2022). Genome sequencing variations in the *Octodon degus*, an unconventional natural model of aging and Alzheimer’s disease. Front. Aging Neurosci. 14:894994. doi: 10.3389/fnagi.2022.894994, PMID: 35860672 PMC9291219

[ref39] Inestrosa N. C. Reyes A. E. Chacón M. A. Cerpa W. Villalón A. Montiel J.. (2005). Human-like rodent amyloid-beta-peptide determines Alzheimer pathology in aged wild-type *Octodon degu*. Neurobiol. Aging 26, 1023–1028. doi: 10.1016/j.neurobiolaging.2004.09.016, PMID: 15748782

[ref40] Jekl V. Diplomate ECZM (Small Mammal) (2021). “23—degus” in Ferrets, rabbits, and rodents. eds. Quesenberry K. E. Orcutt C. J. Mans C. Carpenter J. W. (Philadelphia, PA: W.B. Saunders), 323–333.

[ref41] Jekl V. Hauptman K. Jeklova E. Knotek Z. (2011a). Dental eruption chronology in degus (*Octodon degus*). J. Vet. Dent. 28, 16–20. doi: 10.1177/089875641102800103, PMID: 21696123

[ref42] Jekl V. Hauptman K. Knotek Z. (2011b). Diseases in pet degus: a retrospective study in 300 animals. J. Small Anim. Pract. 52, 107–112. doi: 10.1111/j.1748-5827.2010.01028.x, PMID: 21265850

[ref43] Kumazawa-Manita N. Hama H. Miyawaki A. Iriki A. (2013). Tool use specific adult neurogenesis and synaptogenesis in rodent (*Octodon degus*) hippocampus. PLoS One 8:e58649. doi: 10.1371/journal.pone.0058649, PMID: 23516527 PMC3596278

[ref44] Lee T. M. (2004). *Octodon degus*: a diurnal, social, and Long-lived rodent. ILAR J. 45, 14–24. doi: 10.1093/ilar.45.1.14, PMID: 14752204

[ref45] Lee S. J. Liu T. Chattoraj A. Zhang S. L. Wang L. Lee T. M.. (2009). Posttranscriptional regulation of pineal melatonin synthesis in *Octodon degus*. J. Pineal Res. 47, 75–81. doi: 10.1111/j.1600-079X.2009.00690.x, PMID: 19538336 PMC2837936

[ref46] Lidhar N. K. Thakur A. David A.-J. Takehara-Nishiuchi K. Insel N. (2021). Multiple dimensions of social motivation in adult female degus. PLoS One 16:e0250219. doi: 10.1371/journal.pone.0250219, PMID: 33882104 PMC8059823

[ref47] Long C. V. (2007). Vocalisations of the degu *Octodon Degus*, a social caviomorph rodent. Bioacoustics 16, 223–244. doi: 10.1080/09524622.2007.9753579

[ref48] Long C. Ebensperger L. (2010). Pup growth rates and breeding female weight changes in two populations of captive bred degus (*Octodon degus*), a precocial caviomorph rodent. Reprod. Domest. Anim. 45, 975–982. doi: 10.1111/j.1439-0531.2009.01470.x, PMID: 19497026

[ref49] Márquez N. I. Fernández-Aburto P. F. Deichler A. Perales I. Letelier J.-C. Marín G. J.. (2024). Chilean brush-tailed mouse (*Octodon degus*): a diurnal precocial rodent as a new model to study visual receptive field properties of superior colliculus neurons. *bioRxiv*. Available at: 10.1101/2024.03.25.586655. [Epub ahead of preprint] PMC13413104 39705673

[ref50] Masters C. L. Bateman R. Blennow K. Rowe C. C. Sperling R. A. Cummings J. L. (2015). Alzheimer’s disease. Nat. Rev. Dis. Prim. 1:15056. doi: 10.1038/nrdp.2015.56, PMID: 27188934

[ref51] McMurray C. (2024). How inbreeding almost tanked an up-and-coming model of Alzheimer’s disease. Transmitter. Available at: 10.53053/rocr8999

[ref52] Meserve P. L. Glanz W. E. (1978). Geographical ecology of Small mammals in the northern Chilean arid zone. J. Biogeogr. 5, 135–148. doi: 10.2307/3038168

[ref53] Mota-Rojas D. Marcet-Rius M. Freitas-de-Melo A. Muns R. Mora-Medina P. Domínguez-Oliva A.. (2021). Allonursing in wild and farm animals: biological and physiological foundations and explanatory hypotheses. Animals (Basel) 11:3092. doi: 10.3390/ani11113092, PMID: 34827824 PMC8614478

[ref54] Muñoz-Pedreros A. Yáñez J. (2000). Mamíferos de Chile. Rev. Chil. Hist. Nat. 74, 731–733. doi: 10.4067/S0716-078X2001000300021

[ref55] Nishi M. Steiner D. F. (1990). Cloning of complementary DNAs encoding islet amyloid polypeptide, insulin, and glucagon precursors from a new world rodent, the degu, *Octodon degus*. Mol. Endocrinol. 4, 1192–1198. doi: 10.1210/mend-4-8-1192, PMID: 2293024

[ref56] Okanoya K. Tokimoto N. Kumazawa N. Hihara S. Iriki A. (2008). Tool-use training in a species of rodent: the emergence of an optimal motor strategy and functional understanding. PLoS One 3:e1860. doi: 10.1371/journal.pone.0001860, PMID: 18365015 PMC2268009

[ref57] Opazo J. C. Soto-Gamboa M. Bozinovic F. (2004). Blood glucose concentration in caviomorph rodents. Comp. Biochem. Physiol. A 137, 57–64. doi: 10.1016/j.cbpb.2003.09.007, PMID: 14720591

[ref58] Palacios A. G. Lee T. M. (2013). Husbandry and breeding in the *Octodon degu* (Molina 1782). Cold Spring Harb. Protoc. 2013, 350–353. doi: 10.1101/pdb.prot073577, PMID: 23547155

[ref59] Polis B. Samson A. O. (2024). Addressing the discrepancies between animal models and human Alzheimer’s disease pathology: implications for translational research. J. Alzheimers Dis. 98, 1199–1218. doi: 10.3233/JAD-240058, PMID: 38517793

[ref60] Popović N. Baño-Otálora B. Rol M. Á. Caballero-Bleda M. Madrid J. A. Popović M. (2009). Aging and time-of-day effects on anxiety in female *Octodon degus*. Behav. Brain Res. 200, 117–121. doi: 10.1016/j.bbr.2009.01.001, PMID: 19162080

[ref61] Popović N. Madrid J. A. Rol M. Á. Caballero-Bleda M. Popović M. (2010). Barnes maze performance of *Octodon degus* is gender dependent. Behav. Brain Res. 212, 159–167. doi: 10.1016/j.bbr.2010.04.005, PMID: 20385170

[ref62] Quirici V. Castro R. A. Ortiz-Tolhuysen L. Chesh A. S. Burger J. R. Miranda E.. (2010). Seasonal variation in the range areas of the diurnal rodent *Octodon degus*. J. Mammal. 91, 458–466. doi: 10.1644/08-MAMM-A-337.1, PMID: 22328788 PMC3277432

[ref63] Sensini F. Inta D. Palme R. Brandwein C. Pfeiffer N. Riva M. A.. (2020). The impact of handling technique and handling frequency on laboratory mouse welfare is sex-specific. Sci. Rep. 10:17281. doi: 10.1038/s41598-020-74279-3, PMID: 33057118 PMC7560820

[ref64] Spear G. S. Caple M. V. Sutherland L. R. (1984). The pancreas in the degu. Exp. Mol. Pathol. 40, 295–310. doi: 10.1016/0014-4800(84)90047-9, PMID: 6144570

[ref65] Steffen J. Krohn M. Paarmann K. Schwitlick C. Brüning T. Marreiros R.. (2016). Revisiting rodent models: *Octodon degus* as Alzheimer’s disease model? Acta Neuropathol. Commun. 4:91. doi: 10.1186/s40478-016-0363-y, PMID: 27566602 PMC5002178

[ref66] Švara T. Gombač M. Poli A. Račnik J. Zadravec M. (2020). Spontaneous tumors and non-neoplastic proliferative lesions in pet degus (*Octodon degus*). Vet. Sci. 7:32. doi: 10.3390/vetsci7010032, PMID: 32183187 PMC7158670

[ref67] Tan Z. Garduño B. M. Aburto P. F. Chen L. Ha N. Cogram P.. (2022). Cognitively impaired aged *Octodon degus* recapitulate major neuropathological features of sporadic Alzheimer’s disease. Acta Neuropathol. Commun. 10:182. doi: 10.1186/s40478-022-01481-x, PMID: 36529803 PMC9761982

[ref68] Tokimoto N. Okanoya K. (2004). Acoustic repertoires of the degus: categorization by the acoustic characteristics and vocal contexts. Proceeding of the Psychological and Physiological Acoustics Committee of the Acoustic Society of Japan. 171–175.

[ref69] Valdivia G. Ardiles A. O. Idowu A. Salazar C. Lee H.-K. Gallagher M.. (2023). mGluR-dependent plasticity in rodent models of Alzheimer’s disease. Front. Synaptic Neurosci. 15:1123294. doi: 10.3389/fnsyn.2023.1123294, PMID: 36937569 PMC10017879

[ref70] Vega-Zuniga T. Medina F. S. Fredes F. Zuniga C. Severín D. Palacios A. G.. (2013). Does nocturnality drive binocular vision? Octodontine rodents as a case study. PLoS One 8:e84199. doi: 10.1371/journal.pone.0084199, PMID: 24391911 PMC3877236

[ref71] Woods C. A. Boraker D. K. (1975). Octodon degus. Mamm. Species 67, 1–5. doi: 10.2307/3503820, PMID: 38532229

[ref72] Zampieri G. Cabrol L. Urra C. Castro-Nallar E. Schwob G. Cleary D.. (2024). Microbiome alterations are associated with apolipoprotein E mutation in *Octodon degus* and humans with Alzheimer’s disease. iScience 27:27. doi: 10.1016/j.isci.2024.110348, PMID: 39148714 PMC11324989

